# Intrinsic vs. extrinsic motivation in high school physical education: Which fuels adolescent achievement emotions better?

**DOI:** 10.1371/journal.pone.0327393

**Published:** 2025-06-30

**Authors:** Mustafa Enes Işıkgöz

**Affiliations:** Department of Rectorate, Sakarya University, Sakarya, Türkiye; University of Tartu, ESTONIA

## Abstract

The purpose of this study was to examine the relationship between the intrinsic, extrinsic, and amotivation levels of high school students toward physical education and their emotions of achievement. The study was carried out in the fall semester of the 2024–2025 academic year, with 1399 students studying at various high schools in Turkey. Participants’ motivation levels were assessed using the “Motivation for Participation in Physical Education Lesson Scale,” and their achievement emotions were evaluated with the “Achievement Emotions Scale for Physical Education.” A cross-sectional design was used in the study and data were analysed using structural equation modelling. The findings showed intrinsic motivation strongly related to positive achievement emotions and negatively to negative ones. Extrinsic motivation was weakly correlated with positive achievement emotions and not significantly with negative ones. Amotivation negatively correlated with positive achievement emotions and positively correlated with negative ones. Students’ motivation and emotions are influenced by how well their basic psychological needs are met, according to self-determination and psychological needs theories. The support of intrinsic motivation in physical education is essential for their achievement emotions and well-being. Teachers must promote autonomy, competence, and relatedness in learning environments. Future studies should test these findings in various cultural contexts using various data sources and longitudinal designs. Exploring technological and cultural influences on motivation will add valuable information to the literature.

## Introduction

Physical education is characterized as a multidimensional and interdisciplinary field of learning that supports the physical, cognitive, social and affective development of adolescents. In this process, motivation plays an important role as a fundamental psychological element that determines students’ active participation, performance and long-term exercise habits. Adolescence in particular is a sensitive developmental phase characterized by complex socioemotional dynamics such as identity development, the need for social acceptance and the desire for independence [1]. Recent studies show that adolescents’ motivation for physical education is not limited to academic performance, but is also closely linked to psychological well-being, self-efficacy beliefs and life satisfaction [2–4]. The sedentary lifestyle that has emerged with the acceleration of digitalization has a negative impact on adolescents’ participation in physical activities, which further increases the importance of motivational processes [5,6]. In this context, understanding and supporting adolescents’ motivational processes in physical education has become an important area of research and practice, not only from an educational perspective but also from a public health perspective.

This multifaceted role of motivation in adolescent development is discussed in detail in educational psychology on the basis of self-determination theory (SDT) [7,8]. SDT examines motivation in two basic dimensions: intrinsic and extrinsic. Intrinsic motivation refers to individuals engaging in an activity in accordance with their own inner satisfaction, pleasure and search for meaning. This type of motivation is driven by the individual’s natural curiosity, intrinsic interest and desire for personal development, and the individual feels pleasure in the activity itself regardless of external rewards. Extrinsic motivation, on the other hand, describes the situation in which an individual performs activities to obtain extrinsic rewards (e.g., grades, financial gain, social recognition) or to avoid punishment [8–10]. Motivation, another important concept in SDT, refers to a situation in which an individual is neither intrinsically nor extrinsically motivated to perform an activity [11]. This situation is usually associated with the individual being unable to make a meaningful connection between their behavior and its consequences or believing that they have no control over the consequences of their actions [12].

Current research suggests that intrinsic motivation has multidimensional and positive effects on the developmental processes of young people in physical education. For example, Claver et al [13] have shown that autonomous motivation predicted by the satisfaction of basic psychological needs (including autonomy, competence and relatedness) leads to positive outcomes such as increased self-efficacy, a stronger sense of autonomy and an enhanced sense of belonging in the classroom. Similarly, the study by Vasconcellos et al [14] found that students with high intrinsic motivation were more likely to participate in physical activities and that higher motor performance increased the likelihood that they would maintain regular exercise habits into adulthood. Basic psychological needs theory (BPNT) emphasizes that satisfying students’ needs for autonomy, competence and relatedness increases intrinsic motivation and thus emotions of accomplishment [10,15,16]. Consequently, it is crucial for PE teachers to create learning environments that support students’ basic psychological needs, provide autonomy and promote positive social relationships [14,17].

However, the sedentary lifestyle brought about by the digital age and the influence of social networks on body image are important factors influencing young people’s motivation for physical education. Idealized body images and posts on social media platforms related to physical appearance can direct adolescents’ motivation for physical education towards extrinsic factors (e.g., social acceptance, receiving likes) and negatively influence the development of intrinsic motivation [18,19]. Against this background, it is important for the effectiveness of physical education to determine the types of motivation that promote adolescents’ emotions of achievement in physical education and to investigate the interactions between these types. Recent studies have shown that intrinsic motivation has lasting and profound effects on physical education participation and academic performance [14,20], whereas extrinsic motivation generally produces short-term performance gains, but this effect is not sustainable in the long term. Furthermore, current literature often emphasizes that motivation and achievement emotions depend on the extent to which students’ basic psychological needs are met and how good the learning environment is [21–23].

This study uses a Turkish youth sample to examine how intrinsic and extrinsic motivations are related to achievement emotions in physical education using a structural equation model. In the national context, recent research has emphasized the need for valid and reliable instruments to assess achievement emotions in physical education, as demonstrated by Ceylan and Karlı’s [24] adaptation of the Achievement Emotions Questionnaire for Physical Education (AEQ-PE) into Turkish. However, comprehensive modeling approaches that simultaneously consider both motivation and emotions are still rare in Turkey. Recently, international studies have increasingly focused on the interplay between motivation and achievement emotions using advanced statistical methods. For example, using structural equation modeling, Fierro-Suero et al [21,22] demonstrated that teacher support for basic psychological needs predicts students’ autonomous motivation and positive emotions, which in turn influence academic performance and future intention to be physically active. Similarly, studies have shown that motivational climate and autonomy support are important predictors of motivation and emotional outcomes in physical education [25–29].

Given the observed decline in physical activity and growing motivational problems among adolescents in the post-pandemic period [27], this study provides an evidence-based foundation for the development of educational strategies and intervention programs. The results should help physical education teachers to develop effective interventions to promote students’ intrinsic motivation, e.g., by supporting autonomy and competence, and to improve positive emotions of accomplishment. Therefore, this study makes a unique contribution to the national and international literature by providing concrete recommendations to improve the quality of physical education practice.

### Conceptual and theoretical framework

#### Self-determination theory and types of motivation.

Self-determination theory (SDT) is a comprehensive psychological theory developed by Deci and Ryan [7–10] that focuses on explaining the motivational processes that drive individual behavior. SDT considers motivation in two main dimensions, intrinsic and extrinsic, and emphasizes that the quality of motivation is closely related to the degree to which a person’s basic psychological needs (autonomy, competence and relatedness) are satisfied. According to this theory, the more these needs are satisfied, the more self-determined (self-directed) and sustainable forms of motivation arise.

Intrinsic motivation means that a person engages in an activity because of their natural interest, enjoyment and personal fulfillment [10]. In the context of physical education, intrinsically motivated students engage in physical activities because of intrinsic factors such as curiosity to learn, desire to develop skills, or enjoyment of exercise [3,30]. Research shows that intrinsic motivation is positively associated with long-term physical activity habits, academic performance and psychological well-being [14,20]. For example, the meta-analysis by Vasconcellos et al [14] found that students with high intrinsic motivation were significantly more likely to participate in physical activities and develop their motor skills better. In addition, [30] reported that intrinsic motivation increases students’ positive attitudes towards physical education and their participation in class.

Extrinsic motivation occurs when a person’s behavior is driven by extrinsic factors such as external rewards (grades, praise, bonuses) or the avoidance of punishment [7–10]. However, SDT does not conceptualize extrinsic motivation as a single, unitary construct, but rather as a continuum of regulatory styles that differ in degree of internalization and self-determination. These subtypes include: External regulation, in which behavior is driven solely by external rewards or punishments (e.g., participating in gym class to get a good grade or avoid criticism); Introjective regulation, in which actions are driven by internal pressures such as guilt, anxiety, or the desire for social approval (e.g., participating in activities to avoid guilt or gain teacher approval); Identified regulation, in which the person consciously values the behavior and recognizes its personal significance (e.g., integrated regulation, the most autonomous form of extrinsic motivation, in which the behavior is fully aligned with one’s values and identity (e.g., physical activity because it aligns with one’s self-concept as a healthy person) [8].

These subtypes are classified according to the degree of internalization of extrinsic motivation, with integrated regulation being the most internalized and enduring form [10]. Extrinsic motivation in physical education can increase participation in the short term. For example, students may participate in order to receive grades, praise from their teachers or acceptance from their classmates. However, the sustainability and emotional impact of these different forms of extrinsic motivation can vary [52–54]. More controlled forms (external and introjected regulation) are often associated with less positive emotions and lower engagement, whereas more autonomous forms (identified and integrated regulation) can promote more positive emotional experiences and lasting participation [8]. This distinction may explain why extrinsic motivation as a broad category showed only a weak association with achievement emotions in the present study.

If extrinsic motivation is not differentiated by subtype, the positive effects of the more autonomous forms may be diluted by the less adaptive effects of the controlled forms. However, the sustainability of this type of motivation is limited and can hinder the development of intrinsic motivation in the long term. Particularly in adolescence, extrinsic factors such as grade anxiety or the pursuit of social acceptance have been shown to weaken students’ intrinsic motivation and lead to negative emotions such as apathy or course anxiety [14,16,30].

In SDT, amotivation describes a situation in which a person is neither intrinsically nor extrinsically motivated to perform an activity [31]. This can lead to students being reluctant to participate in physical education, questioning the purpose of the activities or believing that their own efforts have no impact on the outcome. Recent studies have shown that student motivation is related to low self-efficacy, poor academic performance and negative attitudes towards physical activity [11,32,33]. Current literature emphasizes that promoting intrinsic motivation in physical education promotes students’ participation in physical activities, psychological well-being and the development of lifelong healthy habits [20,34]. Therefore, it is crucial for physical education teachers to adopt student-centered and autonomy-supportive approaches that support students’ basic psychological needs with regard to the development of intrinsic motivation.

#### Basic psychological needs theory (BPNT) and physical education.

Basic Psychological Needs Theory (BPNT) was developed by Deci and Ryan [7] as one of the subtheories of Self-Determination Theory (SDT). BPNT states that for mental health and optimal functioning of the individual, three psychological needs must be met that are considered universal and fundamental. Autonomy, Competence, and Relatedness [8]. Meeting these needs increases the individual’s intrinsic motivation, supports the sustainability of their behaviour, and psychological well-being [20,35]. Physical education, as an area that supports the physical, social, and emotional development of adolescents, provides a favourable environment for the fulfilment of basic psychological needs. The need for autonomy refers to the emotion of being able to control one’s own behaviour and make decisions [8,12]. To promote autonomy in physical education, students should have a say in the choice of activities, flexible and student-centred teaching approaches should be adopted, and teachers should support students with motivating and constructive feedback [36,37]. Research shows that autonomy support significantly increases adolescents’ intrinsic motivation and long-term engagement in physical education [34,38]. Learning environments created with autonomy support are also emphasised to strengthen students’ positive attitudes toward the course and their intentions toward physical activity [39–41].

The need for competence reflects a person’s belief that they can be successful in an activity and that they are competent [7]. To promote the emotions of competence in physical education, it is important to set tasks with a level of difficulty that matches the level of students’ ability, to boost their self-confidence through positive and constructive feedback, and to show a supportive attitude in case of failure [33,42]. The literature has found that competence emotions have a strong positive correlation with perceptions of performance in physical education, participation in physical activities, and overall self-efficacy [2,32,43]. Recent studies suggest that supporting efficacy emotions is the key to developing lifelong positive attitudes toward physical activity [4,44]. The need for relatedness refers to an individual’s desire to form social relationships and belong to a group [8,10]. To satisfy this need in physical education, teamwork and cooperative games should be encouraged, a positive classroom environment should be created among students, and communication between teachers and students should be strengthened [30,45]. Research shows that satisfying the need for connection positively influences adolescents’ attitudes toward physical education, emotions of belonging, and social participation [14,16,33]. It is also emphasised that social support and positive peer relationships increase students’ motivation to participate in physical activity [46].

Basic psychological needs theory (BPNT) suggests that inhibiting basic psychological needs can have negative effects on the individual. For example, an overly controlling attitude from teachers can lead to a lack of autonomy, resulting in demotivation in students [17,31]. Repeated failure experiences and inadequate feedback can lead to damaged competence emotions and weakened self-efficacy beliefs [42]. Social exclusion or peer bullying, on the other hand, can have a negative effect on students’ participation in physical education and overall psychological well-being, as the need for connectedness is not fulfilled [11,46]. BPNT provides an effective and holistic framework in the field of physical education. BPNT-based interventions have been shown to increase physical activity levels, psychological well-being, and positive attitudes toward the course [38,47,48]. Studies conducted in Turkey also show that satisfaction of basic psychological needs positively influences students’ attitudes toward physical education, their academic performance, and their motivation for lifelong physical activity. The adaptation and validation of BPNT-related measurement tools for Turkish students further support the importance of these needs in the local context [49–51]. These findings suggest that the support of basic psychological needs in physical education is a crucial element for the academic and psychosocial development of students.

#### Control-value theory (CVT).

Control-value theory (CVT) is a contemporary theory of motivation developed by Pekrun et al. [52] and Pekrun [53] that focusses on explaining individuals’ emotions of achievement in an academic context. According to CVT, students’ emotions of achievement are shaped by two basic cognitive appraisal processes. The first is control beliefs, i.e., an individual’s perception that he or she can influence the outcome of a task; the second is value attribution, i.e., an individual’s assessment of the personal importance of the task [52–54]. These two assessments determine the type and intensity of emotions experienced by students. CVT classifies achievement emotions as positive (e.g., joy, pride, hope) and negative (e.g., fear, shame, sorrow) and emphasises that these emotions have a direct influence on academic performance, motivation, and learning processes [53,55].

Physical education provides a learning environment in which not only physical skills are gained, but also intense emotional experiences are gained. CVT suggests that students’ emotional experiences in physical education play a crucial role in shaping their motivation and participation. Recent research highlights that positive affective states, such as enjoyment derived from physical activity [56], pride following skill mastery [57,58], and hope for future achievement [52,53], act as significant drivers of engagement. On the contrary, negative emotions, including performance anxiety [59], shame related to perceived failure [60], and boredom with repetitive activities, can serve as substantial barriers to participation. Longitudinal studies have reinforced these findings, showing that positive emotional experiences in physical education predict sustained physical activity beyond compulsory schooling [61,62]. In particular, Simonton and Garn [58] found that pride mediates the relationship between skill development and continued participation in sports. Similarly, Simonton et al. [59] reported that performance anxiety is a significant predictor of avoidance behaviours among adolescents. These emotional dynamics are particularly pronounced during adolescence, a period marked by increased sensitivity to social evaluation, which underscores the importance of considering developmental factors in physical education programming [63].

According to CVT, students’ emotions about their performance in physical education are shaped by two basic cognitive appraisals. Belief in control and value attribution. Belief in control triggers positive emotions such as pride and joy when the perception of control is high, e.g., “I can do this activity”, and negative emotions such as fear and shame when the perception of control is low, e.g., “I will fail at this activity”. On the other hand, attribution of values leads to increased motivation and positive emotions when the student perceives the PE lesson as important and meaningful, and negative emotions such as boredom and apathy when the lesson is perceived as unnecessary or unimportant [52–54]. In fact, Frenzel et al. [64] findings show that students’ control beliefs and value attributions in physical education strongly predict their emotional responses to the lesson and their long-term engagement.

In recent years, increasing research has been conducted on professional development in the field of physical education. International studies have shown that control beliefs and value attributions in physical education significantly influence students’ emotional responses to instruction, motivation, and participation in physical activities [58,65,66]. In recent studies conducted in Turkey, the value students place on physical education has been linked to positive attitudes toward the course, and some research suggests that these attitudes may be more pronounced among female students [67,68]. Furthermore, studies show that teachers’ supportive attitudes toward students strengthen their control beliefs and value attributions, which in turn have a positive effect on their emotions of achievement and class participation [69]. To strengthen students’ control beliefs based on CVT, physical education teachers can provide performance experiences by setting tasks with graduated levels of difficulty and strengthening students’ perceptions of self-efficacy through motivating and constructive feedback [58,59]. To strengthen value attribution, strategies such as emphasising the importance of the course in daily life, making connections to a healthy lifestyle, and selecting activities (e.g., dance, yoga, or traditional sports) that match students’ interests can be used [65]. Such practices contribute to the development of positive emotions of achievement in physical education and long-term motivation for physical activity.

In summary, conceptual and theoretical approaches show that adolescents’ motivation and emotions of achievement toward physical education are formed in a multidimensional and dynamic structure. SDT and BPNT show that the intrinsic and extrinsic motivations of students differ according to the degree of fulfilment of their basic psychological needs. Intrinsic motivation is more strongly and sustainably related to participation in physical education, academic performance, and student psychological well-being; extrinsic motivation is more related to short-term performance gains, but its long-term sustainability remains limited. Furthermore, motivation is related to students’ reluctance to participate in class and emotions of underachievement. Control value theory suggests that students’ emotions of achievement in physical education are closely linked to the nature of their motivation. In particular, it emphasises that intrinsic motivation is more related to positive emotions of achievement, such as joy, pride, and hope, while extrinsic motivation is more related to negative emotions of achievement, such as fear, shame, and sorrow.

**Fig 1:**
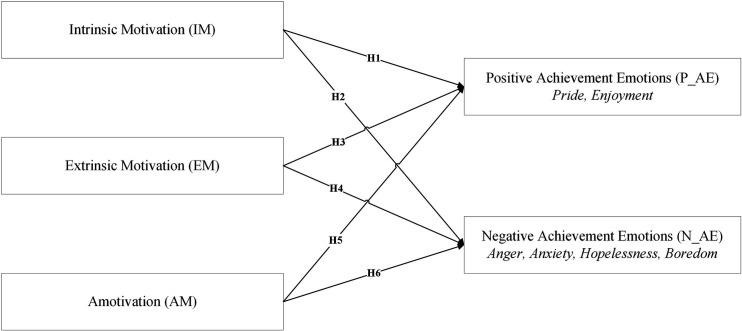
The proposed structure of the study.

Within this framework, the types of motivation that students experience in physical education affect the quality and intensity of their emotions about achievement. In this study, which aims to provide a holistic understanding of the motivational processes that promote adolescents’ emotions of achievement in high school physical education, the objective was to uncover the motivational processes that best support students’ emotions of achievement. To this end, the model shown in Fig 1 was proposed and the following hypotheses were developed.

H1: Intrinsic motivation is positively and significantly related to positive emotions of achievement.
H2: Intrinsic motivation is negatively and significantly related to negative emotions of achievement.
H3: Extrinsic motivation is positively and significantly related to positive emotions of achievement.
H4: Extrinsic motivation is positively and significantly related to negative emotions of achievement.
H5: Amotivation is negatively and significantly related to positive emotions of achievement
H6: Amotivation is positively and significantly related to negative emotions of achievement.

## Methods

### Research design

This study was conducted using a cross-sectional design to investigate the relationships between intrinsic and extrinsic motivation of high school students for physical education and their achievement emotions. A cross-sectional design is an appropriate method to assess the current state of psychological and educational variables and to analyse the relationships between them [70]. In this study, participants’ motivation levels to participate in physical education and their achievement emotions were measured concurrently and the relationships between these variables were assessed. Structural equation modelling (SEM) was used to test the proposed model. SEM is a statistical technique that enables simultaneous testing of measurement and structural models in multivariate data analysis. This method can be used to test theoretical models with empirical data [71,72]. In this study, the relationships between intrinsic motivation, extrinsic motivation, amotivation, and achievement emotions were analysed using SEM and the fit of the model and the validity of the hypotheses were evaluated.

### Study group

The study group consisted of 1399 students studying at various high schools in Turkey during the fall semester of the 2024–2025 academic year. The age of the participants ranged from 14 to 18 years, with a mean age of 15.95 (SD = 1.22). The gender distribution was approximately 53.0% male and 47.0% female. Participants were in different grade levels, from 9th to 12th grade. The results show that the sample has a balanced structure in terms of age, gender, and grade level. In SEM experiments, the appropriate sample size is important depending on the number of parameters in the model. The literature states that at least 10 times the sample size is recommended for each parameter. In this study, considering a total of 37 items and associated parameters, a sample size of 1399 is considered sufficient for SEM analyses [72,73].

### Instruments

#### Motivation for participation in physical education lesson scale.

In this study, the Motivation to Participate in Physical Education Scale, which was developed by Demir and Cicioğlu [43] as part of a validity and reliability study in Turkish culture, was used to measure motivation to participate in physical education. The scale consists of three subdimensions such as intrinsic motivation (IM; 4 items), extrinsic motivation (EM; 5 items) and amotivation (AM; 4 items) and a total of 13 items. The items in the IM and EM dimensions are rated between 1 (strongly disagree) and 5 (strongly agree), while the items in the AM dimension are rated in the opposite direction. Sample items include ‘I enjoy participating in physical education classes’ for IM, ‘I enjoy showing off my athletic skills to people around me’ for EM and ‘I am not sure if participating in these classes is beneficial for me’ for AM. In the original study, the structural validity of the scale was supported by factor analysis and Cronbach’s alpha (α). Internal consistency coefficients were reported as 0.89 for intrinsic motivation, 0.76 for extrinsic motivation, 0.81 for amotivation, and 0.79 for the total scale [43].

In this study, a confirmatory factor analysis (CFA) was performed to assess the fit of the three-factor structure of the scale with the available sample data. Since the scale items have an ordinal Likert-type data structure, the diagonally weighted least squares (DWLS) method was used in the CFA. This method is preferred because it is less dependent on parametric assumptions for ordinal data and provides more accurate results [74]. Furthermore, the DWLS method was found to be suitable as a violation of multivariate normality of the data (Mardia test, p < .001). The analysis results showed that the three-factor model provided an excellent fit with the data (χ²(62) = 56.84, p = 0.66; CFI = 1.00; TLI = 1.00; RMSEA = 0.00 [0.00–0.01]; SRMR = 0.02) [72,75]. All standardised factor loadings (β > 0.70, p < .001) was high and significant. Furthermore, composite reliability (ω) and internal consistency (α) values of 0.93–0.98 (Hair et al., 2018), average variance explanation (AVE) values in the range of 0.76–0.84 [76], and HTMT ratios (<0.85) supported discriminant validity [77].

#### Achievement emotions scale for physical education.

In this study, the six-factor structure of the Achievement Emotions for Physical Education Scale, which was developed by Fierro-Suero et al. [23], and adapted by Işıkgöz [78] through a validity and reliability study in a sample of Turkish high school students, was tested with CFA. The scale comprises a total of 24 items, each consisting of four items with the dimensions of pride, joy, anger, fear, hopelessness, and boredom. The items have a five-point Likert structure ranging from 1 (strongly disagree) to 5 (strongly agree). For the Pride dimension of the questionnaire: I am proud to participate in physical education; for the Fun dimension: When I participate in physical education, I feel good when I follow the teacher’s suggestions; for the anger dimension: I feel anger boiling up inside me during physical education; for the fear dimension: I am afraid of saying/doing something wrong in physical education class and prefer not to say/do anything; for the dimension of hopelessness: I have lost hope that I can carry out the activities in the physical education class effectively; and for the dimension of boredom: I am bored of physical education.

In this study, a CFA was conducted to assess the fit of the six-factor structure of the scale with the available sample data. Since the data did not meet the assumption of multivariate normality (Mardia test, p < .001) and the scale elements were ordinal, the DWLS estimation method was used in the CFA [74]. The fit indices of the six-factor model (χ²(237) = 210.77, p = 0.88; CFI = 1.00; TLI = 1.00; RMSEA = 0.01 [0.00–0.01]; SRMR = 0.02) revealed an excellent fit with the data [72,75]. All standardised factor loadings (β > 0.70, p < .001), they were significant and high [73]. The composite reliability (ω) and internal consistency (α) values of the scale were in the range of 0.96–0.98, indicating high structural internal consistency. Furthermore, the AVE values ranged from 0.87 to 0.96, supporting convergent validity [76]. The HTMT ratios (< 0.85) confirmed that the factors were dissociated [77].

### Ethical approval and consent to participate

This study was approved by the Ethics Committee for Scientific Research and Publications of Mardin Artuklu University (Decision No. E-79906804-050.06.04-87290, dated March 2, 2023). Since the participants were under 18 years of age, informed consent was obtained from both the students and their parents via an online consent form.

### Procedure

The data for this study was collected using the Google Forms platform due to its efficiency, ease of access for participants and time savings. The online survey consisted of two parts. The first section of the study collected demographic data from the participants. The subsequent section contained scale items relevant to the objectives of the study. The recruitment period for this study was from 21/10/2024–25/11/2024.

To ensure compliance with ethical standards, a comprehensive description of the study was provided to participants at the beginning of the study. Informed consent was obtained from participants via an online form. Parents and students were provided with a comprehensive information sheet detailing the study protocol and were asked to give their consent by checking a specific box before proceeding with the survey. The online consent served as written documentation that the participants agreed to participate in the study. The forms indicated that participation was voluntary, that the data would be used for scientific purposes only, that personal information would be kept confidential, and that participants could withdraw from the study without penalty. Access to the remaining sections of the survey was restricted until both consent boxes were checked. The survey link was distributed via social media platforms (e.g., WhatsApp) and email to PE teachers in the relevant secondary schools, and the teachers passed it on to the relevant students.

### Data analysis

All statistical analyses were performed with SPSS (version 27.0) and Jamovi (version 2.5.6). Descriptive statistics (means, standard deviations, skewness, kurtosis, and 95% confidence intervals) were calculated to summarise the demographic characteristics and scale scores of the participants. To examine the measurement properties of the scales, CFA was performed. The CFA was conducted using the DWLS estimation method, which is appropriate for ordinal Likert-type data and when the assumption of multivariate normality is not met [74]. The fit of the model was evaluated using several fit indices, including the chi-square statistic (χ²), comparative fit index (CFI), the Tucker-Lewis index (TLI), the approximate root mean square error (RMSEA), and the standardised root mean square residual (SRMR). CFI and TLI values >0.95, RMSEA <0.06, and SRMR <0.08, indicate a good fit. Furthermore, a ratio of chi-square to degrees of freedom (χ²/df) less than 3 is often considered indicative of an acceptable model fit [72,75]. Internal consistency was evaluated using Cronbach’s alpha (α), with values of 0.70 or greater considered acceptable. Additionally, AVE was calculated to assess convergent validity, with values above 0.50 considered acceptable [76]. Discriminant validity was evaluated using the HTMT, with values below 0.85 indicating adequate discriminant validity [77].

## Results

Table 1 presents the descriptive statistics of the results of the sub-dimensions of the scale related to the participants’ emotions of achievement and motivation to participate in physical education, with a 95% confidence interval. According to the results of the analysis, the “pride” sub-dimension of positive emotions of achievement had the highest mean score (M = 13.55, 95% CI [13.24, 13.86], SD = 5.91), followed by the “enjoyment” sub-dimension (M = 12.94, 95% CI [12.95, 13.49], SD = 6.68). Although the mean values of the sub-dimensions of the negative emotions of achievement were close to each other, “boredom” had the lowest mean value (M = 13.00, 95% CI [12.72, 13.27], SD = 5.28). When examining the sub-dimensions of motivation to participate in physical education, they were categorized as “Intrinsic motivation” (M = 12.89, 95% CI [12.58, 13.19], SD = 5.77), “Extrinsic motivation” (M = 13.39, 95% CI [13.06, 13.73], SD = 6.47) and “Amotivation” (M = 11.83, 95% CI [11.62, 12.04], SD = 4.06). The skewness values calculated to assess the assumption of a normal distribution of the data were between −0.09 and 0.97 and the kurtosis values were between −0.74 and 0.97. The fact that the values are within ±2 indicates that the data are generally close to the normal distribution [79].

**Table 1:** Results of the descriptive statistics for the values obtained from the scales.

Variable Group	Subscale	M (95% CI)	SD	Skewness	Kurtosis
PE Achievement Emotions	Pride	13.55 (13.24, 13.86)	5.91	−0.20	−1.67
	Enjoyment	12.94 (12.95, 13.49)	6.68	−0.34	−1.64
	Anger	13.22 (12.74, 13.29)	5.28	−0.23	−1.53
	Anxiety	13.02 (12.74, 13.29)	5.24	−0.25	−1.52
	Hopelessness	13.11 (12.82, 13.40)	5.57	−0.21	−1.58
	Boredom	13.00 (12.72, 13.27)	5.28	−0.20	−1.54
PE Participation Motivation	Intrinsic	12.89 (12.58, 13.19)	5.77	0.38	−1.80
	Extrinsic	13.39 (13.06, 13.73)	6.47	0.97	−0.74
	Amotivation	11.83 (11.62, 12.04)	4.06	−0.09	−1.46

*Note.* The CI of the mean assumes sample means follow a t-distribution with N – 1 degrees of freedom

Table 2 shows the results of the Spearman correlation analysis of the relationship between motivation level and achievement emotions in physical education. All correlations between the variables were statistically significant (p < .001). The results show significant positive relationships between intrinsic motivation and pride (r = 0.73, p < .001) and enjoyment (r = 0.55, p < .001). An increase in intrinsic motivation contributes to an increase in pride and joy, which are positive emotions of achievement. These two positive emotions of achievement were also found to be strongly correlated (r = 0.89, p < .001).

**Table 2:** Results of the correlation analysis between the emotions in physical education and the level of motivation.

	1	2	3	4	5	6	7	8
1. Intrinsic Motivation	—							
2. Extrinsic Motivation	0.62^***^	—						
3. Amotivation	−0.66^***^	−0.46^***^	—					
4. Pride	0.73^***^	0.57^***^	−0.62^***^	—				
5. Enjoyment	0.55^***^	0.46^***^	−0.46^***^	0.89^***^	—			
6. Anger	−0.69^***^	−0.53^***^	0.63^***^	−0.81^***^	−0.71^***^	—		
7. Anxiety	−0.68^***^	−0.53^***^	0.63^***^	−0.79^***^	−0.70^***^	0.92^***^	—	
8. Hopelessness	−0.69^***^	−0.51^***^	0.64^***^	−0.82^***^	−0.72^***^	0.92^***^	0.89^***^	—
9. Boredom	−0.68^***^	−0.51^***^	0.63^***^	−0.78^***^	−0.70^***^	0.89^***^	0.91^***^	0.89^***^

*** *p < .001*

In addition, significant and negative correlations were found between intrinsic and extrinsic motivation and negative emotions of achievement. The results show that there are significant relationships between anger and intrinsic motivation (r = −0.69, p < .001), between anxiety and intrinsic motivation (r = −0.68, p < .001) and between anger and extrinsic motivation (r = −0.53, p < .001). On the other hand, a low level of motivation is also associated with an increase in the negative achievement emotions of anger, anxiety, hopelessness and boredom. For example, there are significant correlations between hopelessness and amotivation (r = 0.64, p < .001) and boredom and amotivation (r = 0.63, p < .001).

In this study, the relationships between intrinsic motivation (IM), extrinsic motivation (EM) and amotivation (AM) and positive (P_EM) and negative (N_EM) emotions of achievement were analyzed using structural equation modeling (SEM). Since the data were of Likert type and did not meet the assumption of multiple normal distribution, the DWLS estimation method was preferred for the analysis [74]. In the model, “Pride (Prd)” and “Joy (Enj)” were defined as positive achievement emotions (P_EM); “Anger (Ang)”, “Anxiety (Anx)”, “Hopelessness (Hpl)” and “Boredom (Brd)” were defined as negative achievement emotions (N_EM).

The decision to group these specific emotions into broader “positive” and “negative” constructs is based on both theoretical and empirical grounds. According to the Control Value Theory (CVT) of achievement emotions [52–55], emotions experienced in academic settings can be meaningfully categorized as positive or negative based on their valence and their typical impact on motivation and learning outcomes. Previous research has also supported the use of higher-order positive and negative emotion factors in academic and physical education contexts, as these groupings capture common variance and facilitate model parsimony [53,58]. However, it is recognized that each individual emotion may have unique antecedents and consequences. Therefore, additional analyzes were conducted to examine the effects of motivation types on each emotion separately.

The goodness-of-fit indices of the measurement model showed that the model was an excellent fit to the data (χ²(613) = 584.35, p = 0.79; CFI = 1.00; TLI = 1.00; RMSEA = 0.01; SRMR = 0.02) [72,75]. The factor loadings of all latent variables in the observed variables were high and significant (β > 0.81, p < .001). Furthermore, the values of the internal consistency coefficients (α > 0.92) and the explained average variance (AVE > 0.75) on the scales indicate that the measurement model has a high degree of reliability and validity [73,76]. These results show that the measurement model is both structurally and statistically sound and reliable.

**Fig 2:**
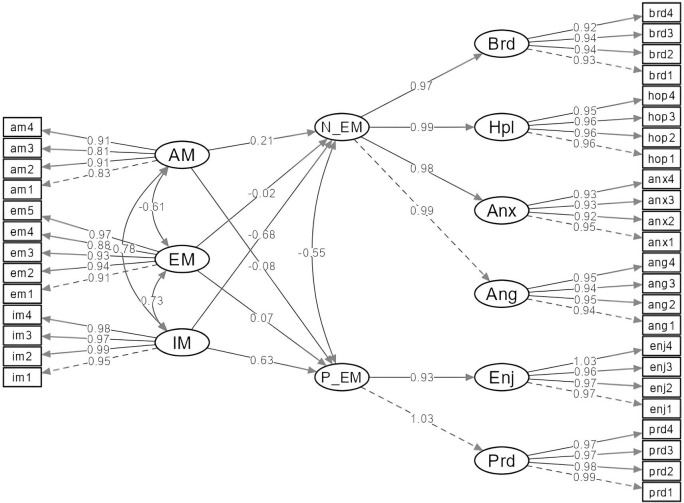
Structural model flow chart.

Following the measurement model, the proposed structural model (Fig 1) and the hypotheses of the study are tested. In the structural model, IM, EM and AM are considered as exogenous variables, while P_EM and N_EM are considered as endogenous variables. The goodness of fit indices of the structural model show that the model provides an excellent fit to the data (χ²(613) = 584.35, p = 0.12; CFI = 1.00; TLI = 1.00; RMSEA = 0.01; SRMR = 0.02) [72,75]. The high internal consistency (α = 0.98 for IM; α = 0.97 for EM; α = 0.92 for AM) and AVE (0.94 for IM; 0.87 for EM; 0.75 for AM) of the model support a solid foundation for the structural model [73,76,80]. The results of both the measurement model and the structural model indicate that the model is valid and reliable. The path diagram of the structural model is shown in Fig 2 and the parameter estimates are listed in Table 3.

**Table 3:** Path coefficients of the structural model between sources of motivation and emotions of achievement.

				*95% CI*					
**Pred**	**Dep**	**B**	**SE**	**Lower**	**Upper**	**β**	**z**	**p**	**R²**
**IM**	P_EM	0.82	0.03	0.76	0.87	0.63	28.97	< .001	.55
**EM**		0.10	0.02	0.06	0.14	0.07	4.99	< .001	
**AM**		−0.15	0.03	−0.22	−0.09	−0.08	−4.55	< .001	
**IM**	N_EM	−0.66	0.02	−0.70	−0.62	−0.68	−34.15	< .001	.74
**EM**		−0.02	0.01	−0.05	0.01	−0.02	−1.52	.130	
**AM**		0.29	0.02	0.25	0.33	0.21	12.84	< .001	

IM: Intrinsic motivation, EM: Extrinsic motivation, AM: Amotivation, P_EM: Positive emotions, N_EM: Negative emotions

The results of Table 3 show that intrinsic motivation (IM) has a strong and significant positive relationship with positive emotion of achievement (P_EM) (β = 0.63, p < .001). This finding supports that students with higher levels of IM experience more intense P_EM. Extrinsic motivation (EM) has a weak but statistically significant positive relationship with P_EM (β = 0.07, p < .001). This limited role of EM on P_EM suggests that extrinsic factors can partially predict positive emotions toward achievement, but not as decisively as IM. Amotivation (AM) showed a negative and significant relationship with P_EM (β = −0.08, p < .001). This result indicates that individuals with a high level of AM experience lower P_EM.

IM showed a strong and significant negative relationship with negative achievement emotions (N_EM) (β = −0.68, p < .001). This result suggests that individuals with high IM experience less N_EM. EM showed a very weak negative relationship with N_EM (β = −0.02, p = .130). This suggests that EM does not play a significant role in attenuating negative emotions. AM showed a positive and significant relationship with N_EM (β = 0.21, p < .001). This result supports the notion that individuals with higher levels of AM experience more N_EM. In the model, IM, EM, and AM together explain 55% of the variance of P_EM (R² = 0.55) and 73% of the variance of N_EM (R² = 0.73). These results show that IM is primarily a major predictor of the emergence of achievement emotions, AM has a function of decreasing positive and increasing negative emotions, and EM has a limited relationship with achievement emotions.

In this study, an alternative structural model was tested in which each achievement emotion (pride, enjoyment, anger, anxiety, hopelessness and boredom) was analyzed as a separate outcome variable. This approach allowed us to examine whether the associations between intrinsic motivation (IM), extrinsic motivation (EM) and amotivation (AM) varied across emotions rather than across the general positive/negative categories. The model showed excellent goodness-of-fit indices (χ²(593) = 437.10, CFI = 0.99, TLI = 0.99, RMSEA = 0.05, SRMR = 0.05) [72,75]. As shown in Table 4, results indicated that IM showed strong positive associations with pride (β = 0.68, p < .001) and enjoyment (β = 0.54, p < .001), along with strong negative associations with hopelessness (β = −0.67, p < .001) and boredom (β = −0.68, p < .001). IM was also significantly and negatively associated with anger (β = −0.64, p < .001) and anxiety (β = −0.67, p < .001).

**Table 4:** Alternative model: achievement emotions as separate outcomes.

						*β 95% CI*					
**Pred.**	**Dep.**	**B**	**SE**	**β**	**Lower**		**Upper**	**z**	**p**
IM	Pride	0.85	0.03	0.68	0.63		0.73	25.16	<.001
	Enjoyment	0.68	0.03	0.54	0.49		0.59	20.63	<.001
	Anger	−0.64	0.03	−0.64	−0.69		−0.59	−23.17	<.001
	Anxiety	−0.68	0.03	−0.67	−0.73		−0.62	−23.80	<.001
	Hopelessness	−0.70	0.03	−0.67	−0.72		−0.62	−24.25	<.001
	Boredom	−0.66	0.03	−0.68	−0.73		−0.62	−23.75	<.001
EM	Pride	0.12	0.02	0.09	0.05		0.12	5.02	<.001
	Enjoyment	0.08	0.02	0.05	0.02		0.09	3.34	<.001
	Anger	−0.06	0.02	−0.05	−0.09		−0.02	−2.88	0.01
	Anxiety	−0.03	0.02	−0.02	−0.06		0.01	−1.23	0.22
	Hopelessness	−0.01	0.02	−0.01	−0.04		0.03	−0.43	0.67
	Boredom	0.00	0.02	0.00	−0.03		0.04	0.23	0.82
AM	Pride	−0.11	0.04	−0.06	−0.10		−0.02	−2.66	0.01
	Enjoyment	−0.20	0.04	−0.11	−0.15		−0.07	−4.96	<.001
	Anger	0.29	0.03	0.20	0.16		0.25	8.74	<.001
	Anxiety	0.28	0.03	0.20	0.15		0.24	8.40	<.001
	Hopelessness	0.32	0.03	0.21	0.17		0.26	9.38	<.001
	Boredom	0.28	0.03	0.20	0.15		0.25	8.38	<.001

IM: Intrinsic motivation, EM: Extrinsic motivation, AM: Amotivation

EM had a weak but statistically significant positive associations with pride (β = 0.09, p < .001) and enjoyment (β = 0.05, p < .001). EM was also significantly and negatively associated with anger (β = −0.05, p < 0.05), but no significant links were found with anxiety, hopelessness, and boredom (p > 0.05). In contrast, AM showed positive associations with anger, anxiety and boredom (β = 0.20, p < .001) and hopelessness (β = 0.21, p < .001). AM was also significantly and negatively associated with pride (β = −0.06, p < 0.05) and enjoyment (β = −0.11, p < .001).

Overall, these results support the validity of grouping emotions into positive and negative categories for reasons of parsimony and theoretical clarity, but also show that the strength of associations between motivation types and specific emotions can vary. Therefore, it may be beneficial for future research to examine both higher order models and discrete emotions to capture the complexity of students’ emotional experiences in physical education.

In addition, a supplementary analysis was conducted to investigate whether gender moderates the relationships between types of motivation and achievement emotions. The results of the cross-group SEM analysis are presented in Table 5. The results show that the model fit well for both the male and female groups (χ²(1232) = 0.80, CFI = 0.99, TLI = 0.99, SRMR = 0.02, RMSEA = 0.00).

**Table 5:** Multigroup SEM gender comparison: path coefficients and chi-square tests.

Pred.	Dep.	Males (β)	Females (β)	Δχ²	p
IM	P_EM	0.51^***^	0.61^***^	4.31	0.03
EM		0.06^***^	0.07^***^	8.92	0.01
EM		−0.17^***^	−0.17^***^	0.12	0.73
IM	N_EM	−0.70^***^	−0.66^***^	16.10	<.001
EM		−0.02	−0.02	4.13	0.04
EM		0.22^***^	0.18^***^	17.01	<.001

IM: Intrinsic motivation, EM: Extrinsic motivation, AM: Amotivation, P_EM: Positive emotions, N_EM: Negative emotions, ^***^p < .001, β = Standardized path coefficient; Δχ² = Chi-square difference (from constraints score test), p = significance value for group difference.

The comparison of the path coefficients revealed significant gender-specific differences for several associations. In particular, the association between intrinsic motivation and positive emotions was β = 0.51 for males and β = 0.61 for females, with this difference reaching statistical significance (Δχ² = 4.31, p < 0.05). Similarly, the strength of the relationship between intrinsic motivation and negative emotions was β = −0.70 for males and β = −0.66 for females, which was also significant (Δχ² = 16.10, p < .001). The correlation between extrinsic motivation and positive emotions was β = 0.06 for males and β = 0.07 for females (Δχ² = 8.92, p < 0.05) and for negative emotions β = −0.02 for both males and females (Δχ² = 4.13, p < 0.05), with both paths showing significant gender differences.

In addition, the relationship between amotivation and negative emotions was β = 0.22 for males and β = 0.18 for females, and this difference was also statistically significant (Δχ² = 17.01, p < .001). These results suggest that the relationships between types of motivation and achievement emotions may vary by gender. In contrast, the relationships between amotivation and positive emotions did not differ significantly by gender (Δχ² = 0.12, p < 0.05). All path coefficients and the results of the chi-square difference test for the group comparisons are listed in Table 5.

According to the test results of the hypotheses in Table 6, intrinsic motivation has a significant positive relationship with positive emotions of achievement (H1) and a significant negative relationship with negative emotions of achievement (H2). Extrinsic motivation has a weak positive relationship with positive emotions of achievement (H3) and no significant relationship with negative emotions of achievement (H4). Amotivation was found to have a significant negative relationship with positive emotions of achievement (H5) and a significant positive relationship with negative emotions of achievement (H6).

**Table 6:** Summary of the results of the hypothesis test.

Hypothesis	Results
**H1:** Intrinsic motivation is positively and significantly related to positive emotions of achievement.	Supports-positive
**H2:** Intrinsic motivation is negatively and significantly related to negative emotions of achievement.	Supports-negative
**H3:** Extrinsic motivation is positively and significantly related to positive emotions of achievement.	Supports-positive (weak)
**H4:** Extrinsic motivation is positively and significantly related to negative emotions of achievement.	Rejected
**H5:** Amotivation is negatively and significantly related to positive emotions of achievement	Supports-negative
**H6:** Amotivation is positively and significantly related to negative emotions of achievement.	Supports-positive

## Discussion

This study provides a comprehensive and up-to-date perspective on the literature on physical education by examining the relationship between high school students’ motivational types and their emotions of achievement in physical education. The results suggest that physical education is a multidimensional learning environment that promotes not only physical development but also the emotional and psychological well-being of students. These findings once again confirm that, within the framework of self-determination theory (SDT) and basic psychological needs theory (BPNT), students’ motivational and emotional experiences depend on the extent to which their basic psychological needs are met [14,35,42]. It is important to note that these processes are not isolated from the broader cultural context in which students are embedded. In Turkey, cultural norms such as respect for authority, collectivist values, and emphasis on academic achievement may influence both the expression and internalization of motivation in physical education [81,82]. Thus, due to societal expectations, familial influences, and the hierarchical structure of schools, Turkish students are more prone to introjected or external regulation, which may influence their emotional responses to PE activities.

The first and second hypotheses tested in the study (H1 and H2) revealed that intrinsic motivation has a significant and strong positive relationship with positive achievement emotions and a significant negative relationship with negative achievement emotions; these hypotheses were fully supported. This finding suggests that students whose autonomy, competence, and relationship needs are met, as predicted by SDT, develop higher intrinsic motivation and positive emotions in physical education [7,8,10,14,16]. Furthermore, intrinsic motivation contributes to academic achievement by promoting students’ emotions of autonomy and competence and increasing participation not only in class but also in extracurricular physical activities [22,40,41,83]. It is emphasized that a mastery-oriented learning climate promotes intrinsic motivation, whereas a performance-oriented climate can have a negative impact on motivation by increasing students’ stress and anxiety levels [84,85].

In addition, current research confirms that teacher support and the provision of autonomy play an important role in satisfying students’ basic psychological needs and maintaining motivation [38]. However, in the Turkish context, the traditional teacher-centered approach and the high value placed on teacher authority may limit the opportunities for autonomy support, which could interfere with the development of students’ intrinsic motivation [86]. This is in line with SDT research in other non-Western cultures, which has shown that autonomy-supportive practices are less common or interpreted differently in collectivist societies, but still play a crucial role in promoting positive motivational and emotional outcomes [87,88].

This study also explored the relationships between motivational types and discrete emotions, revealing nuanced associations beyond the general positive/negative categories. Consistent with the literature emphasizing the analysis of discrete emotions [59,89], intrinsic motivation was strongly associated with pride and enjoyment and negatively associated with hopelessness, boredom, anger, and anxiety. Extrinsic motivation showed a weaker positive association with pride and enjoyment and a negative association with anger. Amotivation was associated with increased anger, anxiety, boredom and hopelessness. These findings suggest that both higher-order models and discrete emotions should be investigated to better understand students’ emotional experiences in physical education [52,58,90].

The third hypothesis (H3) stated that extrinsic motivation is related to positive emotions of achievement. However, the results showed that this relationship was statistically significant but weak and the hypothesis was only partially supported. The fourth hypothesis (H4) assumed that extrinsic motivation has a significant relationship with negative emotions of achievement. However, this hypothesis could not be confirmed and extrinsic motivation was not significantly related to negative emotions. Consistent with the motivational continuum approach of SDT, it appears that internalised forms of extrinsic motivation, in particular (e.g., identified and integrated regulation), may increase engagement in the short term, but have limited emotionally enduring and deepening effects if basic psychological needs are not adequately met [35,91,92]. Furthermore, reward-based approaches have been shown to undermine students’ needs for autonomy, competence, and relatedness and may prevent the internalisation of motivation in the long term [10,35,36,42]. Some recent studies also suggest that extrinsic rewards can support intrinsic motivation by creating motivational synergies under certain conditions [93]. However, the general trend is that extrinsic motivation is not sufficient to create lasting emotional satisfaction and can lead to apathy by weakening students’ emotions of self-determination. Therefore, a balance should be struck in the use of extrinsic motivational tools in physical education and autonomy-enhancing approaches should be prioritized to support students’ long-term intrinsic motivation [35,94,95]. In Turkey, the strong influence of family expectations and societal norms may reinforce external and introjected forms of motivation, as students often feel pressure to meet parental or teacher standards [96]. This cultural emphasis on external validation may help to explain the weak relationship between extrinsic motivation and positive emotions observed in this study. Similar findings have been reported in SDT research from other non-Western contexts, where external regulation is more prominent and may be associated with increased anxiety or decreased well-being [97].

The fifth hypothesis (H5) predicted that motivation is negatively related to positive emotions of achievement and the results supported this hypothesis. The sixth hypothesis (H6) predicted that amotivation would be positively related to negative emotions of achievement, and this hypothesis was also strongly supported. In the context of SDT and BPNT, it is known that in the case of amotivation, students’ basic psychological needs are not met, resulting in low self-efficacy, low academic achievement, and negative emotional outcomes [11,30,98]. For example, a study by Wang et al. [99] reported that students with high levels of amotivation were more likely to be absent from physical education classes and their participation rate in physical activities was significantly lower. Negative emotional effects such as helplessness, low self-esteem, and depressive symptoms are more common in students with low motivation [21,22,100]. This has a negative effect on students’ physical health and overall quality of life. The current literature shows that supportive teaching practices and autonomy-supportive learning environments are effective in reducing the negative effects of lack of motivation [22,98]. Satisfying students’ basic psychological needs (autonomy, competence, relatedness) contributes to reducing amotivation by increasing motivation and leading to positive emotional outcomes. Furthermore, understanding the underlying causes of amotivation enables the development of effective interventions that promote student participation and emotional well-being in physical education [30,58]. In the Turkish education system, where academic achievement is highly valued and physical education may be undervalued, students who do not perceive PE as important or relevant may be more likely to be amotivated. This is consistent with the findings of SDT in other collectivist societies, where the perceived value of PE and congruence with cultural priorities can significantly influence students’ motivation and emotional experience [3,87].

The study also found that gender moderates the relationships between motivation and emotions. The link from intrinsic motivation to positive emotions was stronger in females, while the negative link to negative emotions was more pronounced in males. Gender also influenced the associations between extrinsic motivation and positive emotions and between amotivation and negative emotions. These findings suggest that gender-specific approaches are needed to improve motivation and emotional well-being in physical education [27,30].

The results of the study are also consistent with the Control Value Theory (CVT). According to CVT, students’ emotions about their performance in physical education are closely related to their control beliefs and the value they attribute to the course [52–55]. Students with high intrinsic motivation have a high perception of control that they can achieve something in the course and attribute a high value to the course, which contributes to an increase in positive emotions (joy, pride). In contrast, a low perception of control and a low attribution of value in highly amotivated students lead to negative emotions such as anxiety, helplessness, and boredom [58,59,65]. Current research in the context of physical education emphasises the crucial role of teacher behaviour and teaching strategies in motivating students. In particular, autonomy-supportive teaching approaches – practices such as recognising students’ right to choice, providing logical explanations, and understanding the student perspective – have been shown to increase students’ intrinsic motivation and reduce negative emotional experiences [36]. Similarly, a study by Jang et al. [101] found that peer learning and collaborative activities improve both motivational and affective outcomes in physical education.

These findings suggest that it is important for physical education teachers to apply student-centred and participatory approaches in their classroom interactions to increase student interest and emotional well-being.The current literature also draws attention to the influence of technology on motivational processes in physical education. In particular, exergue-based learning environments (e.g., virtual reality-enriched physical activities) have been reported to increase interest and engagement among low-motivated students [44,102,103]. However, more empirical research is needed on the long-term effects and sustainability of such technological applications. Furthermore, recent studies investigating the influence of cultural factors on motivational processes suggest that extrinsic sources of motivation (e.g., family approval or societal expectations) may be more crucial in non-Western societies [44,104]. In Turkey, the collectivist orientation and the importance placed on group harmony and respect for authority may influence students’ responses to motivational strategies and their emotional experiences in physical education. Thus, students may be more responsive to teacher-directed activities and less likely to express personal preferences, which may influence both their motivation and emotional outcomes [96,104]. SDT research in non-Western cultures, including Turkey, has emphasized the need to adapt autonomy-supportive practices to local cultural values, suggesting that culturally sensitive approaches are essential for promoting motivation and well-being in diverse educational settings [99,105]. These findings indicate that the cultural context needs to be considered when developing motivational strategies in physical education. In different cultural contexts, students’ sources of motivation and their responses to instructional strategies can be very different.

### Limitations and suggestions for future research

Although this study provides valuable information about the relationships between high school students’ motivation types and their achievement emotions in physical education, several limitations should be considered. First, the study was conducted with a Turkish sample only, which may limit the generalizability of the findings to other cultural and educational contexts. Replication of similar studies in other countries and cultural settings is important to test the robustness of these findings. Second, the data were based on self-report, which may lead to bias due to social desirability or incomplete self-reflection. In addition, the exclusive use of self-report data raises the possibility of general method variance. To mitigate this, efforts were made to ensure anonymity and to use clear, concise wording. However, future research should consider using multiple data collection methods, such as observations, teacher and parent reports, or qualitative interviews, to improve validity and reduce common method variability. Third, the cross-sectional design of the study does not allow conclusions to be drawn about the causality between motivation types and achievement emotions. Longitudinal studies or experimental investigations in future studies would help to clarify causal relationships.

In this study, only gender was examined as a potential moderator; the possible moderating effect of grade level should be investigated in future studies. In addition, this study focused only on students’ individual perceptions without considering environmental factors such as teacher behavior, classroom climate, school policies, or family support, all of which can have a significant impact on students’ motivational and emotional experiences. It is recommended that such variables at multiple levels and from the environment be included in future research. While practical recommendations are made, these remain broad. Future studies should examine specific examples of autonomy-supportive practices or instructional strategies that PE teachers could employ. For example, teachers could provide students with choices in activities, offer rationales for tasks, acknowledge students’ feelings, and encourage student initiative [7]. SDT-based intervention programs, such as the one described by Reeve [36], offer relevant models for promoting intrinsic motivation and positive emotions in the school environment. In addition, the inclusion of strategies to promote connectedness, such as cooperative learning activities and peer support, could promote a more positive emotional climate in physical education. To further enhance the development and testing of intervention programs, future studies could benefit from the use of motivational behavior classification systems, such as the one developed by Ahmadi et al [106]. This classification system could be very useful to develop and test the effectiveness of an intervention program aimed at improving need-supportive/motivational behaviors in high school physical education classes. Finally, the study did not examine in depth the effects of technological applications or cultural differences on motivational processes. Examining the effects of exergame-based interventions or culturally specific motivational strategies represents a promising direction for future research. Addressing these limitations through the use of diverse samples, multiple data sources, longitudinal studies, and consideration of teacher and environmental factors as well as technological and cultural influences will make an important contribution to the physical education literature.

## Conclusions

The results of this study show that adolescents’ emotions of achievement in physical education are largely characterized by intrinsic motivation. Intrinsic motivation strengthens both positive emotions and negative emotions. Extrinsic motivation has only a limited effect, and a lack of motivation has a negative association with emotional well-being. In addition, a lack of motivation negatively influences students’ participation in class and their attitudes towards physical activity, leading to negative academic and emotional outcomes. The overwhelming support for the research hypotheses confirms that intrinsic motivation plays a central role in adolescents’ emotional experiences in physical education. Therefore, it is important for educators to adopt pedagogical approaches that support students’ intrinsic motivation in order to both increase positive emotions related to achievement and contribute to the reduction of negative emotions. Creating supportive and inclusive learning environments that meet students’ basic psychological needs is essential to prevent lack of motivation and promote students’ emotional well-being.
